# Diseases of the Abdomen

**Published:** 1903-01-03

**Authors:** 


### DISEASES OF THE ABDOMEN.

(*Concluded from page* 217.)

Jaundice and hepatic pain have been found associated, by J. Hutchinson, jun.,[11] with floating kidney, while there were no gall-stones present. The symptoms were completely relieved by nephropexy. The obvious deduction was that the floating kidney had been the means of obstructing the flow of bile. The writer cannot find any evidence of a hepato-renal band of peritoneum, as described by Weisker, which can drag upon the neck of the gall bladder. The gastro-hepatic omentum reaches from the transverse fissure of liver to the duodenum, but as it does not extend outwards to the first bend of the duodenum, it is quite free of the kidney. There is nowhere any ridge or band connecting the right kidney with the liver or gall bladder, unless it be of pathological formation. Neither does displacing the right kidney forwards and downwards produce any such fold. Displacement downwards of the kidney will displace also the third part of the duodenum, and stretch the common bile-duct. The gall bladder is displaced with sharp kinking of the cystic duct, the vertical part of the duodenum is twisted and perhaps even the bile duct. This dragging of the duodenum may be the real cause of the gastro-intestinal symptoms so often found associated with floating right kidney. He has also observed a case of glycosuria associated with floating kidney which was relieved by rest in bed and nephropexy. There has been an epidemic of jaundice in the East Falkland Islands, the infectivity of which Foley [12] has had considerable evidence of.

He is inclined to attribute this to influenza, the catarrhal jaundice being a new manifestation of this protean malady.

Professor Schäfer [13] points out the existence within the liver cells of channels which can be injected from the blood vessels. They are too narrow to admit of blood corpuscles, but able to convey the fluid portion of the blood directly to the cell protoplasm. Hay's reaction for bile salts (viz., sulphur sprinkled upon the surface of a fluid containing them sinks easily, owing to the surface tension being reduced), according to Beddard and Pembery [14] is most useful in urine and fœces. The urine must be cold, clear, and free from air bubbles. If the sulphur falls at once then there is an indication of the presence of bile salts to the extent of more than 1 in 10,000. If it does not fall till shaken after 1 minute's delay, then the quantity is 1 in 40,000.

R. Hutchison [15] has examined the opalescent fluid obtained from a case of chiliform ascites in a patient suffering from chronic parenchymatous nephritis. He finds this is due to a proteid which "leaves a phosphorus-containing residue on digestion, presumably a nuclein or paranuclein. Lecithin and mucoid substances could in this case be excluded.

Wace Carlier [16] reports the analysis of two specimens of chyle obtained from the thoracic duct through an accidental wound in the neck of a child aged 10. The first gave—

| | Per cent. |
|---|---|
| Water ... ... ... ... | 92·12 |
| Fat, lecithin, Cholesterin ... ... ... | 4·88 |
| Mineral salts, Proteids, etc. ... ... ... | 3·00 |
| Total solids ... ... ... ... | 7·88 |

The second gave: Sp. grav. 1017·1 at 17° C.
Analysis:—

| | Per cent. |
|---|---|
| Water ... ... ... ... | 92·519 |
| Fat, lecithin, Cholesterin ... ... ... | 2·820 |
| Proteid ... ... ... ... | 3·840 |
| Fibrin and other organic substances ... ... ... | 0·391 |
| Total organic solids ... ... | 7·051 |
| NaCl ... ... ... ... | 0·155 |
| Other salts... ... ... ... | 0·275 |
| Total mineral matter ... ... | 0·430 |
| Total solids ... ... ... | 7·481 |

Two enzymes have been found in the spleen by Hedin and Rowland.[17] One acting in an acid medium, digesting proteid as far as any enzyme known. And a second acting in an alkaline medium. Both are probably contained in the cells of the spleen. That an acid can act within a living cell is maintained by comparison with certain infusoria with their acid-digesting fluid vacuoles, and phagocytic leucocytes.

Adrenalin has been isolated by Takamine.[18] He finds it to have the approximate formula $C_{10}H_{15}NO_3$, though this has not been properly worked out. It consists of light, white, microscopic crystals with bitter taste and leaving a numb feeling on the tongue. It is stable if dry, soluble in hot water more readily than in cold, forming a slightly alkaline solution. It forms salts with acids and alkaline hydroxides. These solutions oxidise in air. It gives a green colour with ferric chloride, a red with iodine. Houghton, of Detroit, finds that a fraction of a drop of aqueous solution of adrenalin or its salts, in a strength of 1 in 50,000, blanches the normal conjunctiva within one minute. It is a powerful hæmostatic, and intravenous injection leads to great rise of blood-pressure. Adrenalin is over a thousand times stronger than fresh gland. It is used already as an astringent, hæmostatic in inflammatory conditions, and as a cardiac stimulant. It is non-irritating, non-cumulative, and has no injurious properties. It is an antidote in some ways to morphine and opium poisoning, and can be used with advantage in circulatory failure, in preventing collapse during anæsthesia, and especially for bloodless work on the eye, ear, nose, and throat. It has a place in the treatment of deafness, hay-fever, nasal hæmorrhage, and various forms of heart disease.

Bayliss and Starling [19] have shown that an increased flow of pancreatic juice induced by the introduction of an acid into the duodenum and jejunum is not due to reflex stimulation. It occurs if the nerve endings are paralysed and destroyed, and after intravenous injection of atropine-sulphate. It must be therefore due to the excitation of gland cells by means of a substance carried in the blood. By the action of ·4 per cent. HCL. on the mucous membrane of the duodenum and jejunum a secrétion having no action upon the pancreas is changed into an agent capable of producing copious pancreatic secretion. The authors think this only one instance of chemical affinity in the body, and that this will open up possibilities of therapeutic control of various chemical functions.

[11] Practitioner, Feb. [12] Brit. Med. Jour., May 8. [13] Med. Rec., March 8. [14] Brit. Med. Jour., March 22. [15] Ibid., March 8. [16] Ibid., July 19, 1902. [17] Ibid., March 8. [18] Scot. Med. and Surg. Jour., Feb., and Lancet, Dec. 21. [19] Brit. Med. Jour., Mar. 8

